# Supplementary figures and images for: Influence of rimonabant treatment on peripheral blood mononuclear cells; flow cytometry analysis and gene expression profiling
Source: PeerJ. 2015 Jun 30;3:e1056. doi: 10.7717/peerj.1056 (PMC4493638; doi:10.7717/peerj.1056)

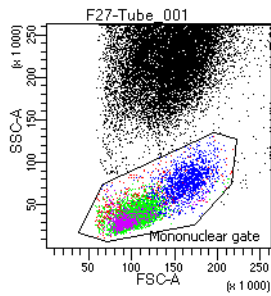

Experiment Name: F27\_001

| Population       | #Events | %Parent | %Total |
|------------------|---------|---------|--------|
| All Events       | 30 000  | ###     | 100.0  |
| Mononuclear gate | 7 523   | 25.1    | 25.1   |
| CD3+             | 4 470   | 59.4    | 14.9   |
| Q1               | 3 023   | 67.6    | 10.1   |
| Q2               | 60      | 1.3     | 0.2    |
| Q3               | 86      | 1.9     | 0.3    |
| Q4               | 1 301   | 29.1    | 4.3    |
| CD3-CD4+         | 1 472   | 19.6    | 4.9    |
| CD19+            | 594     | 7.9     | 2.0    |

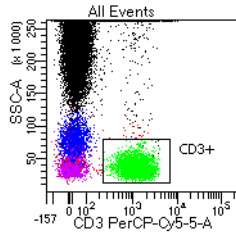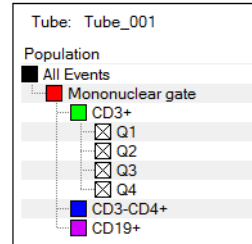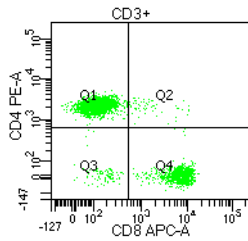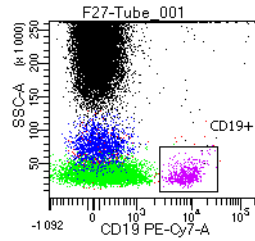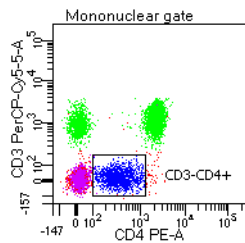

Supplement: Figure S1 — The gating strategy is shown in the dendrogram. First a mononuclear cell gate was set. Within this gate subpopulations of CD3+ T cells, CD19+ B cells and CD3−, CD4+ cells were identified by additional gating. [file peerj-03-1056-s001.pdf]
